# Small Molecule Inhibitors of *Mycobacterium tuberculosis* Topoisomerase I Identified by Machine Learning and In Vitro Assays

**DOI:** 10.3390/ijms252212265

**Published:** 2024-11-15

**Authors:** Somaia Haque Chadni, Matthew A. Young, Pedro Igorra, Md Anisur Rahman Bhuiyan, Victor Kenyon, Yuk-Ching Tse-Dinh

**Affiliations:** 1 Biochemistry PhD Program, Department of Chemistry and Biochemistry, Florida International University, Miami, FL 33199, USA; schadni@fiu.edu; 2 Atomwise Inc., San Francisco, CA 94103, USA; mayoung01@gmail.com (M.A.Y.); victor@atomwise.com (V.K.); 3 Department of Chemistry and Biochemistry, Florida International University, Miami, FL 33199, USA; pigor001@fiu.edu (P.I.); mbhuiyan@fiu.edu (M.A.R.B.); 4 Biomolecular Sciences Institute, Department of Chemistry and Biochemistry, Florida International University, Miami, FL 33199, USA

**Keywords:** tuberculosis, TB, mycobacterium, topoisomerase I, ML-based drug discovery, virtual screening

## Abstract

Tuberculosis (TB) caused by *Mycobacterium tuberculosis* is a leading infectious cause of death globally. The treatment of patients becomes much more difficult for the increasingly common multi-drug resistant TB. Topoisomerase I is essential for the viability of *M. tuberculosis* and has been validated as a new target for the discovery of novel treatment against TB resistant to the currently available drugs. Virtual high-throughput screening based on machine learning was used in this study to identify small molecules that target the binding site of divalent ion near the catalytic tyrosine of *M. tuberculosis* topoisomerase I. From the virtual screening of more than 2 million commercially available compounds, 96 compounds were selected for testing in topoisomerase I relaxation activity assay. The top hit that has IC_50_ of 7 µM was further investigated. Commercially available analogs of the top hit were purchased and tested with the in vitro enzyme assay to gain further insights into the molecular scaffold required for topoisomerase inhibition. Results from this project demonstrated that novel small molecule inhibitors of bacterial topoisomerase I can be identified starting with the machine-learning-based virtual screening approach.

## 1. Introduction

Antimicrobial resistance (AMR) is an urgent global public health problem that must be addressed to avoid a future of facing superbugs that are resistant to all available treatments [1]. AMR has been a serious issue for the treatment of tuberculosis (TB) patients with multi-drug resistant (MDR-) and extensively drug-resistant (XDR-) TB [2] requiring much longer periods of treatment. While newly developed TB drug regimens have been useful for improving the therapy for drug-resistant TB, the emerging resistance to the drugs in these regimens such as bedaquiline, delamanid and pretomanid is a great concern [2,3]. The development of novel drugs to combat drug-resistant TB and AMR in general remains a high priority.

Topoisomerases are master regulators of genome topology. By coupling the cutting and rejoining of DNA accompanied by the passage of DNA through the transient breaks, topoisomerases can remove topological barriers encountered during vital cellular processes including replication, transcription and repair [4,5,6]. Depending on whether topoisomerases cut a single strand or double strand of DNA during catalysis, they are classified as type I or type II topoisomerases, which can then be divided into subfamilies based on similarities in sequence and mechanism [4,7]. Topoisomerases are the targets of many important anticancer and antibacterial drugs used in the clinic [8,9,10]. To date, these topoisomerase-targeting drugs include inhibitors of type IB [11,12] and type IIA [13,14] subfamilies of topoisomerases but not type IA topoisomerases [15]. Bacterial topoisomerase I belonging to the type IA topoisomerase subfamily is essential for the viability of many pathogens [16,17,18] including *M. tuberculosis* [19], the causative agent of TB. *M. tuberculosis* topoisomerase I (MtbTOP1) and gyrase are present as the only type I and type II topoisomerase in this organism. They have been proposed as targets for the discovery of new anti-TB drugs [20]. *M. tuberculosis* gyrase is the target of moxifloxacin, a fluoroquinolone that is part of some of the latest therapeutic regimens [2,21]. MtbTOP1 should be further explored as a validated new target [22] to aid the efforts of countering TB drug resistance.

In the project described here, we utilized machine-learning-based virtual screening to target the active site region of MtbTOP1, including the binding site of divalent ion required for catalytic activity [23,24]. From 96 compounds selected from the virtual screening, the in vitro enzyme activity assay identified a novel inhibitor of MtbTOP1 with IC_50_ of 7.0 µM. We further investigated the commercially available analogs of this top hit.

## 2. Results

### 2.1. Machine-Learning-Based Virtual Screen for Inhibitors of MtbTOP1

**Figure 1:**
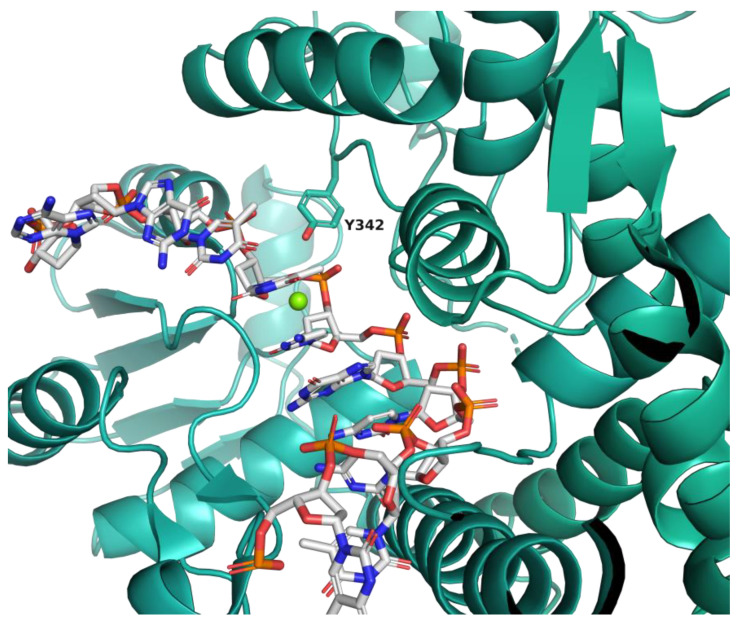
Active site of ssDNA-bound MtbTOP1 (PDB ID: 6CQI) showing the location of catalytic Y342 in teal stick and the catalytic Mg^2+^, superimposed from the Mg^2+^-bound structure 6CQ2, as a green sphere. The virtual screen was performed on this binding site in the absence of the Mg^2+^ ion.

Many inhibitors targeting the DNA binding site of MtbTOP1 have been identified through virtual screening [25,26,27], including machine learning methods [28]. Our previous studies docked and scored molecules in the active site near the catalytic Y342 of 5D5H as no DNA-bound structures were available. The recently available crystal structures of MtbTOP1 with bound ssDNA or ssDNA plus Mg^2+^ (PDB ID: 6CQI and 6CQ2, respectively) were used to target the divalent cation binding site (Figure 1) to screen a library of over 2 million compounds to identify potential binders of MtbTOP1. From the screen, the top-ranked compounds were filtered by structure and calculated physicochemical properties to remove structural alerts, potential PAINS, and compounds with undesirable properties (e.g., LogP > 5, etc.) [29,30,31,32,33,34,35,36,37]. Of the top-ranked molecules, 96 compounds (Table S1) were synthesized and shipped for in vitro screening.

### 2.2. Identification of MtbTOP1 Inhibitors from the Virtual Screening Hits

**Figure 2:**
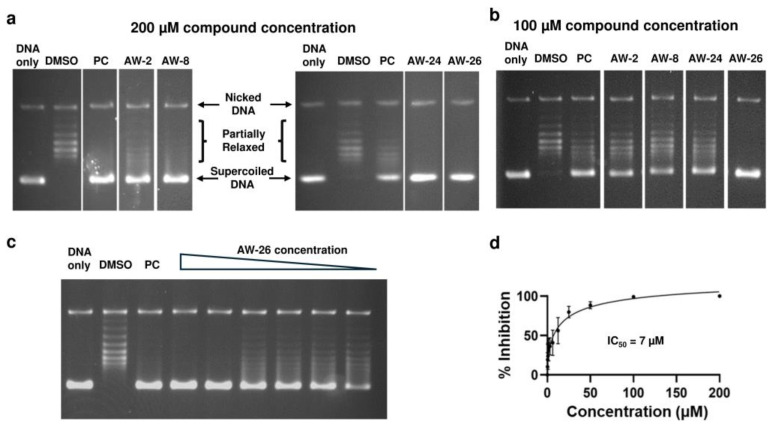
MtbTOP1 inhibitors identified from assays of virtual screening hits for inhibition of relaxation of negatively supercoiled DNA. PC: 8 µM NSC76027 [40] as positive control. (**a**) Compounds found to inhibit close to 100% of MtbTOP1 relaxation activity at 200 µM concentration. The two sets of lanes shown are each from a single gel. (**b**) Testing of inhibition of MtbTOP1 relaxation activity by the identified inhibitors at 100 µM concentration. The lanes shown are from a single gel. (**c**) Inhibition of MtbTOP1 relaxation by 2-fold serial dilutions of compound AW-26 from 200 to 6.25 µM. (**d**) The mean and standard deviation from six repeated experiments were plotted with GraphPad Prism version 8.4.2 to obtain the IC_50_ value of AW-26.

We first assayed the inhibition of MtbTOP1 relaxation activity by the 96 small molecules selected by virtual screening at 200 µM concentration. Four compounds (AW-2, AW-8, AW-24 and AW-26) were found to inhibit close to 100% of the relaxation activity at this concentration (Figure 2a). Among these four compounds (Table 1), only compound AW-26 showed complete inhibition of MtbTOP1 at 100 µM concentration (Figure 2b). The inhibition of MtbTOP1 relaxation activity by AW-26 (Enamine Z15957051) was characterized by further serial dilutions (Figure 2c). The IC_50_ of inhibition was determined to be 7.0 µM (Figure 2d). The compound was reordered from Enamine (Kyiv, Ukraine) and found to have the same potency as the compound supplied by Atomwise (San Francisco, CA, USA).

**Figure 3:**
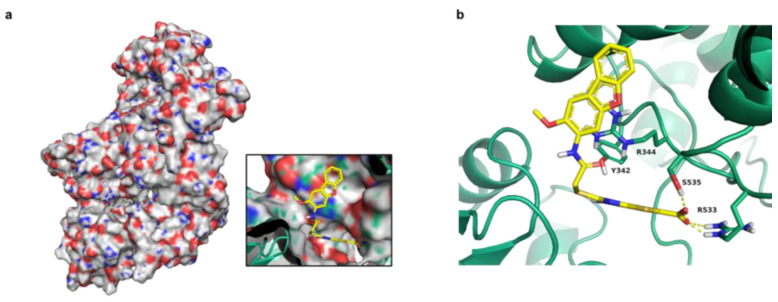
Representative docking pose of AW-26. (**a**) AW-26 docked to MtbTOP1, with the insert zooming in on the active site. (**b**) Hydrogen bonds between docked AW-26 (yellow, dashed lines) and residues R533 and S535 at the active site of MtbTOP1.

Glide SP (Schrödinger, New York, NY, USA) was subsequently employed to generate physics-based poses of the inhibitor ligands identified in the relaxation assay. Multiple ionization states and chiralities for unspecified stereochemistries of AW-2, AW-8, AW-24 and AW-26 were generated and docked to the prepared MtbTOP1 (PDB: 6CQI). No co-crystallized small molecule inhibitors of MtbTOP1, or any closely related structures, are present in the PBD for use in this study. The DNA in 6CQI was removed, similar to the AIM Screen, to expose the binding site. AW-2, AW-8 and AW-26 were docked to the same pocket (Figure 3a) with conserved interactions, including a cation–pi interaction between R344 and aromatic rings of the ligands, with the carboxylate of AW-26 making a hydrogen bond with R533, likely conferring the improved potency (Figure 3b).

**Table 1:** Virtual screening hits found to inhibit MtbTOP1 relaxation activity.

Code (Enamine ID)	Glide Emodel Score [38,39]	Structure
AW-2 (Z87600393)	−63.535	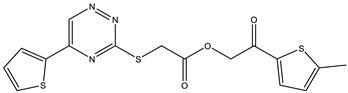
AW-8 (Z113583514)	−69.157	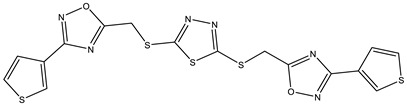
AW-24 (Z57788727)	−69.784	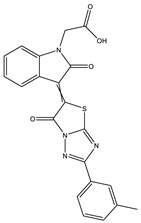
AW-26 (Z15957051)	−70.747	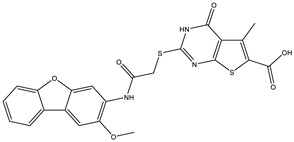

### 2.3. Evaluation of AW-26 Analogs for MtbTOP1 Inhibition

**Figure 4:**
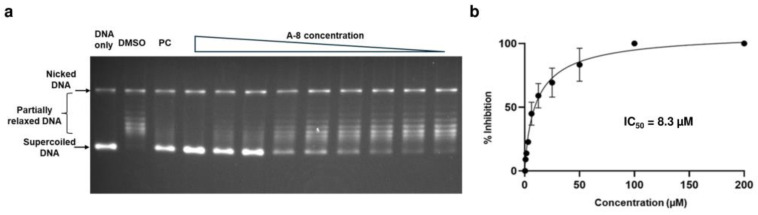
Assay of inhibition of MtbTOP1 relaxation of negatively supercoiled DNA by AW-26 analog A-8. (**a**) Inhibition of MtbTOP1 relaxation by 2-fold serial dilutions of compound A-8 from 200 to 0.8 µM. PC: 8 µM NSC76027 as positive control. (**b**) The mean and standard deviation from three repeated experiments were plotted with GraphPad Prism version 8.4.2 to obtain the IC_50_ value.

Twelve commercially available analogs of AW-26 (Table 2) were purchased from Chembridge (San Diego, CA, USA) and Enamine and tested for the inhibition of MtbTOP1 relaxation activity (Figure 4 and Figure S1). AW-26 was also repurchased from Enamine. Analog A-8 (Chembridge 7984991) was the only evaluated analog that showed a complete inhibition of MtbTOP1 at 100 µM (Figure 4a). The assay of 2-fold serial dilutions of analog A-8 showed that it has an IC_50_ of 8.3 µM (Figure 4b), close to the IC_50_ value of 7.0 µM determined for AW-26. None of the other analogs showed similar potency for the inhibition of MtbTOP1 (Figure S1). Analogs A-1, A-4 and A-11 showed a partial inhibition of MtbTOP1, but the percent inhibition observed was 50% or less when tested at 50 µM (Figure S1).

### 2.4. Evaluation of Inhibitor Selectivity with Human Topoisomerase I Assay

**Figure 5:**
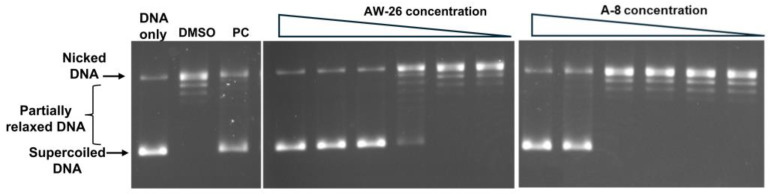
Assay of inhibition of human topoisomerase I relaxation activity by AW-26 and A-8. Two-fold serial dilutions of (AW-26 and A-8 from 200 to 6.25 µM) were tested for inhibition of relaxation of negatively supercoiled DNA by 1 unit of human topoisomerase I. PC: 50 µM myricetin [41,42] as positive control. All lanes shown are from the same gel.

To evaluate the selectivity of inhibitors AW-26 and A-8, they were assayed for the inhibition of the type IB human topoisomerase I relaxation activity. The type IA MtbTOP1 and type IB human topoisomerases I share no sequence homology and have different catalytic residues in the active site [7]. The assay results (Figure 5) showed that the IC_50_ for the inhibition of human topoisomerase I relaxation activity is between 25 and 50 µM for AW-26 and between 50 and 100 µM for A-8.

## 3. Discussion

In this study, we targeted the active site of MtbTOP1, which contains residues important for DNA cleavage and rejoining, and the binding of the divalent ion required for the enzyme relaxation activity as well. Virtual high throughput screening based on machine learning was employed to identify a set of 96 small molecules as candidates for the inhibition of MtbTOP1 activity. Four of the small molecules supplied by Atomwise showed inhibition of MtbTOP1 relaxation activity in enzyme activity assays. The hit rate is 4.2% compared to an average hit rate of 7.6% for the 296 Atomwise academic collaboration targets [29]. The Atomwise AIMS academic collaboration on *M. tuberculosis* gyrase as the target (ID P9WG47) has a hit rate of 0% from testing 94 compounds [29]. It is possible that identifying small molecules capable of inhibiting enzyme interactions including electrostatic interactions with DNA substrate or divalent ion poses a greater challenge than identifying inhibitors that can compete for the binding of most other enzyme or receptor substrates. The 20 Atomwise projects targeting MtbTOP1, gyrase, transcription factors or DNA/RNA-binding proteins that are included in the reported Atomwise AIMS project have an average hit rate of 4.7% (Table S2), demonstrating the opportunity for the improvement of computational methods for use against similar targets.

AW-26 is the only one of the four hits for MtbTOP1 that showed complete inhibition of relaxation activity at 100 µM. Further dose–response measurements showed that it has an IC_50_ of 7.0 µM. To further validate the action of this inhibitor, we identified and obtained 12 commercially available analogs of AW-26 that share some similar elements in the molecular structure. Four of the twelve analogs showed at least partial inhibition of MtbTOP1 relaxation activity. Analogs A-1, A-4 and A-8 possess the thieno-pyrimidine-carboxylic acid moiety while analog A-11 has the methoxy-dibenzofuran moiety present in AW-26. The IC_50_ of inhibition by analog A-8 (8.3 µM) is close to that of AW-26. Further computational studies could potentially improve the potency of the inhibition of MtbTOP1 by identifying the optimal combination of scaffold elements.

In addition to the potency requirements for MtbTOP1 inhibitors, selectivity and whole-cell activity are also needed for the inhibitors to be useful for anti-TB applications. The assay of the inhibition of human topoisomerase I by AW-26 and A-8 showed a better dose–response inhibition of MtbTOP1 than human topoisomerase I by these small molecules, but a greater degree of selectivity would need to be achieved. We could not detect any antibacterial activity for these two compounds against *Mycobacterium smegmatis*. The sequence alignment of MtbTOP1 and *M. smegmatis* topoisomerase I confirmed the high degree of sequence identity (82%), including target site residues (Figure S2), so the compounds AW-26 and A-8 are likely to interact similarly with the two proteins. The lack of whole-cell activity for the two compounds tested could be due to either insufficient potency or lack of cellular entry/retention. For future anti-mycobacterial drug discovery efforts, it would be useful to develop AI tools to predict whole-cell entry/retention to complement the target-based identification of potential inhibitors.

While a number of the previously identified inhibitors of MtbTOP1 may have advantages over AW-26 and A-8 for possessing greater potency against MtbTOP1 and/or whole-cell activity against *M. tuberculosis* and *M. smegmatis* [43], many of these inhibitors are DNA binders or intercalators that can act on cellular DNA or additional DNA binding proteins. These include m-AMSA [27], seconeolitsine [44], Gold(III) complexes [45] and fluoroquinophenoxazine [46]. Some of the MtbTOP1 inhibitors are known to interact with other cellular targets with similar or greater affinity than MtbTOP1 based on the literature and PubChem records. The tricyclic antidepressants identified in previous virtual screening of MtbTOP1 inhibitors [26] bind human serotonin transporters and other proteins [47]. Compound NSC76027 [40] has been identified in HTS to be an inhibitor of *M. tuberculosis* phosphoserine phosphatase [48]. The novel inhibitors AW-26 and A-8 described here have no other reported nucleic acid or protein targets so far according to PubChem records, suggesting the possibility of greater selectivity of MtbTOP1 as a specific target.

## 4. Materials and Methods

### 4.1. Assay of MtbTOP1 Relaxation Activity

Recombinant MtbTOP1 was expressed and purified from *Escherichia coli* host strain C41(DE3) as described [49]. Compounds provided by Atomwise based on the virtual screening results were dissolved in DMSO to make a stock solution of 10 or 20 mM depending on solubility and then diluted in DMSO to the concentration of 8 mM. Inhibition of relaxation activity of MtbTOP1 was analyzed in reaction buffer containing 10 mM Tris-HCl, pH 8.0, 50 mM NaCl, 0.1 mg/mL gelatin and 0.5 mM MgCl_2_. A buffer containing 50% glycerol, 100 mM potassium phosphate pH 7.4 and 0.2 mM EDTA was used for dilution of the enzyme. DMSO or compound from 8 mM solution (0.5 µL) was first added to 9.5 µL reaction buffer containing 20 ng of enzyme followed by the addition of 10 µL of reaction buffer containing 200 ng of supercoiled pBAD/Thio plasmid DNA (purified by CsCl gradient centrifugation) for a final reaction volume of 20 µL. After mixing by gentle vortex, the reaction mixtures were spun down and incubated at 37 °C for 30 min. Then, 4 µL of stop solution (50% glycerol, 50 mM EDTA and 0.5% (*v*/*v*) bromophenol blue) was added to each reaction. An inhibitor of bacterial topoisomerase I (NSC76027) identified from previous virtual screening against the covalent complex of *E. coli* topoisomerase I [40] was included at 8 µM as positive control. The supercoiled DNA substrate was separated from the relaxed DNA topoisomers by electrophoresis in 1% agarose gel with TAE buffer (40 mM Tris-acetate, pH 8.1 and 2 mM EDTA) for 20 h at 25 V. The DNA was stained with ethidium bromide for an hour and then soaked in water for 5 min. Gels were photographed over UV light with the AlphaImager system. The amount of the supercoiled DNA substrate remaining in each reaction was quantified using the AlphaImager software version 1.5.0 to calculate the percentage of inhibition.

### 4.2. Assay of Human Topoisomerase I Relaxation Activity

Recombinant human topoisomerase I was expressed and purified from the *Saccharomyces cerevisiae* strain EKY3 transformed with plasmid pYES2-TOP1 as described [41]. Inhibition was assayed in a reaction buffer containing 10 mM Tris-HCl, pH 8.0, 1 mM EDTA, 150 mM NaCl, 0.1 mM spermidine, 0.1 mg/mL BSA and 5% glycerol. DMSO or compound dilutions in volume of 0.5 µL was first added to 9.5 µL reaction buffer containing 1 unit of enzyme (defined as amount required for complete relaxation of input supercoiled DNA) before the addition of 10 µL of reaction buffer containing 200 ng of supercoiled pBAD/Thio plasmid DNA (purified by CsCl gradient centrifugation) for a final reaction volume of 20 µL. Following mixing by gentle vortex, the reaction mixtures were spun down and incubated at 37 °C for 30 min before the addition of 4 µL of stop solution (6% SDS, 0.6% (*w*/*v*)) bromophenol blue and 40% glycerol. Myricetin (50 µM), previously studied as a human topoisomerase I inhibitor [41,42], was included as a positive control. Gel electrophoresis for separation of the supercoiled DNA substrate from the relaxed DNA topoisomers was conducted as described for the MtbTOP1 relaxation activity assay.

### 4.3. Machine-Learning-Based Molecular Screen for Inhibitors of MtbTOP1

The small molecule Virtual High-Throughput Screen (VHTS) was performed, as previously described using Atomwise’s proprietary machine-learning-based AtomNet^®^ screening platform [29,30,31,32,33,34,35,36,37]. Briefly, the AtomNet neural network utilizes a 3D input layer, then multiple 3D-convolutional and fully connected layers, to assign probabilities of whether a compound will bind the protein target site. The AtomNet global model was trained with a dataset with thousands of protein structures and millions of small molecule ligands. A curated library of over 2 million small molecules (enamine_instock_v200204—containing 2,273,009 small molecules that have been synthesized by Enamine to represent the billions of compounds that are readily accessible for synthesis using the Enamine building blocks) was scored and ranked to identify potential inhibitors of MtbTOP1 using the location of the divalent cation and DNA-binding sites on *M. tuberculosis* topoisomerase I (PDB ID: 6CQI and 6CQ2, respectively) [23] defined by the amino acid residues V23, E24, S25, K28, S45, R46, G47, H48, T110, D111, G112, D113, R114, E115, G116, I119, L160, Y342, R344, A388, H389 and S535.

As in previous studies, physicochemical properties were calculated with ICM (Molsoft), and the top-ranked 200 molecules were reduced to a list of 96 compounds with drug-like properties (e.g., Lipinski’s rule of 5). The 96 selected compounds from the VHTS were purchased for testing at stock concentrations of 10 mmol L^−1^ in DMSO and validated to be ≥85% purity via LC–MS at Mcule.

### 4.4. Docking of MtbTOP1 Inhibitors

Docking of the inhibitors using Glide was described previously [50]. Briefly, the 2.4 A crystal structure of MtbTOP1 (6CQI) was prepared for docking using the protein preparation wizard in Maestro. No waters or DNA were retained in the active site for docking. Up to 16 instances of ligands were prepared using LigPrep in Maestro to investigate different ionization states and stereocenters. The docking grid was created centered around Y342, and the ligands were docked using the SP mode. The resulting poses were sorted by Glide Emodel score to compare the binding mode between isomers.

## Figures and Tables

**Table 2:** Analogs of AW-26 evaluated for MtbTOP1 inhibition with relaxation assays.

Code (Supplier ID)	Glide Emodel Score [38,39]	Structure
A-1 (Enamine) Z15958059	−67.743	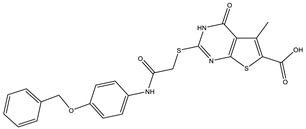
A-2 (Enamine) Z15958225	−71.473	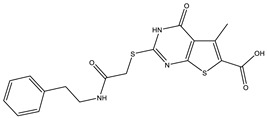
A-3 (Enamine) Z15957449	−73.029	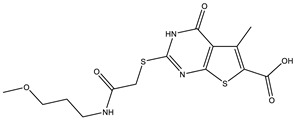
A-4 (Chembridge) 7845718	−75.116	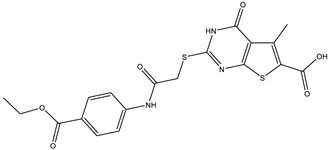
A-5 (Chembridge) 7912129	−57.485	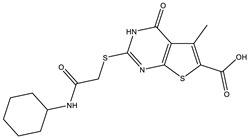
A-6 (Chembridge) 7861220	−61.034	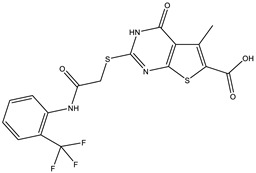
A-7 (Chembridge) 7949754	−59.505	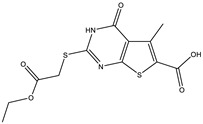
A-8 (Chembridge) 7984991	−73.247	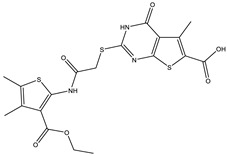
A-9 (Chemibridge) 7964501	−79.611	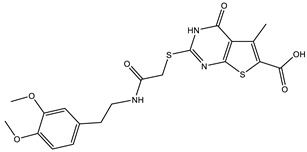
A-10 (Chembridge) 7912130	−72.992	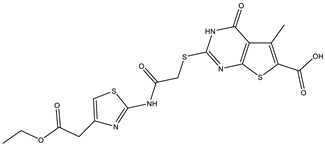
A-11 (Enamine) Z188745626	−70.756	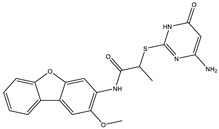
A-12 (Enamine) Z55950797	−66.143	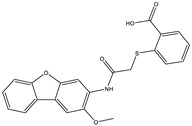

## Data Availability

All data generated or analyzed during this study are included in this published article and its Supplementary Information files.

## Supplementary Materials

The following supporting information can be downloaded at https://www.mdpi.com/article/10.3390/ijms252212265/s1.

## References

[1] Tang K.W.K., Millar B.C., Moore J.E. (2023). Antimicrobial Resistance (AMR). Br. J. Biomed. Sci..

[2] Pedersen O.S., Rudolf F., Johansen I.S., Andersen Å.B., Lillebæk T., Wejse C.M., Dahl V.N. (2024). Drug-resistant tuberculosis is a global cause of concern. Ugeskr. Laeger.

[3] Negi A., Perveen S., Gupta R., Singh P.P., Sharma R. (2024). Unraveling Dilemmas and Lacunae in the Escalating Drug Resistance of Mycobacterium tuberculosis to Bedaquiline, Delamanid, and Pretomanid. J. Med. Chem..

[4] McKie S.J., Neuman K.C., Maxwell A. (2021). DNA topoisomerases: Advances in understanding of cellular roles and multi-protein complexes via structure-function analysis. Bioessays.

[5] Pommier Y., Nussenzweig A., Takeda S., Austin C. (2022). Human topoisomerases and their roles in genome stability and organization. Nat. Rev. Mol. Cell Biol..

[6] Vos S.M., Tretter E.M., Schmidt B.H., Berger J.M. (2011). All tangled up: How cells direct, manage and exploit topoisomerase function. Nat. Rev. Mol. Cell Biol..

[7] Bush N.G., Evans-Roberts K., Maxwell A. (2015). DNA Topoisomerases. EcoSal Plus.

[8] Schreiber A.R., O’Bryant C.L., Kabos P., Diamond J.R. (2023). The emergence of targeted therapy for HER2-low triple-negative breast cancer: A review of fam-trastuzumab deruxtecan. Expert. Rev. Anticancer Ther..

[9] Bjornsti M.A., Kaufmann S.H. (2019). Topoisomerases and cancer chemotherapy: Recent advances and unanswered questions. F1000Research.

[10] Bush N.G., Diez-Santos I., Abbott L.R., Maxwell A. (2020). Quinolones: Mechanism, Lethality and Their Contributions to Antibiotic Resistance. Molecules.

[11] Thomas A., Pommier Y. (2019). Targeting Topoisomerase I in the Era of Precision Medicine. Clin. Cancer Res..

[12] Cinelli M.A. (2019). Topoisomerase 1B poisons: Over a half-century of drug leads, clinical candidates, and serendipitous discoveries. Med. Res. Rev..

[13] Nitiss J.L. (2009). Targeting DNA topoisomerase II in cancer chemotherapy. Nat. Rev. Cancer.

[14] Vann K.R., Oviatt A.A., Osheroff N. (2021). Topoisomerase II Poisons: Converting Essential Enzymes into Molecular Scissors. Biochemistry.

[15] Pommier Y. (2013). Drugging topoisomerases: Lessons and challenges. ACS Chem. Biol..

[16] Suerbaum S., Brauer-Steppkes T., Labigne A., Cameron B., Drlica K. (1998). Topoisomerase I of Helicobacter pylori: Juxtaposition with a flagellin gene (flaB) and functional requirement of a fourth zinc finger motif. Gene.

[17] Yan R., Hu S., Ma N., Song P., Liang Q., Zhang H., Li Y., Shen L., Duan K., Chen L. (2019). Regulatory Effect of DNA Topoisomerase I on T3SS Activity, Antibiotic Susceptibility and Quorum- Sensing-Independent Pyocyanin Synthesis in Pseudomonas aeruginosa. Int. J. Mol. Sci..

[18] García-López M., Megias D., Ferrándiz M.J., de la Campa A.G. (2022). The balance between gyrase and topoisomerase I activities determines levels of supercoiling, nucleoid compaction, and viability in bacteria. Front. Microbiol..

[19] Ahmed W., Menon S., Godbole A.A., Karthik P.V., Nagaraja V. (2014). Conditional silencing of topoisomerase I gene of Mycobacterium tuberculosis validates its essentiality for cell survival. FEMS Microbiol. Lett..

[20] Nagaraja V., Godbole A.A., Henderson S.R., Maxwell A. (2017). DNA topoisomerase I and DNA gyrase as targets for TB therapy. Drug Discov. Today.

[21] Nyang’wa B.T., Berry C., Kazounis E., Motta I., Parpieva N., Tigay Z., Moodliar R., Dodd M., Solodovnikova V., Liverko I. (2024). Short oral regimens for pulmonary rifampicin-resistant tuberculosis (TB-PRACTECAL): An open-label, randomised, controlled, phase 2B-3, multi-arm, multicentre, non-inferiority trial. Lancet Respir. Med..

[22] Ravishankar S., Ambady A., Awasthy D., Mudugal N.V., Menasinakai S., Jatheendranath S., Guptha S., Sharma S., Balakrishnan G., Nandishaiah R. (2015). Genetic and chemical validation identifies Mycobacterium tuberculosis topoisomerase I as an attractive anti-tubercular target. Tuberculosis.

[23] Cao N., Tan K., Annamalai T., Joachimiak A., Tse-Dinh Y.C. (2018). Investigating mycobacterial topoisomerase I mechanism from the analysis of metal and DNA substrate interactions at the active site. Nucleic Acids Res..

[24] Dasgupta T., Ferdous S., Tse-Dinh Y.C. (2020). Mechanism of Type IA Topoisomerases. Molecules.

[25] Sandhaus S., Chapagain P.P., Tse-Dinh Y.C. (2018). Discovery of novel bacterial topoisomerase I inhibitors by use of in silico docking and in vitro assays. Sci. Rep..

[26] Godbole A.A., Ahmed W., Bhat R.S., Bradley E.K., Ekins S., Nagaraja V. (2015). Targeting Mycobacterium tuberculosis topoisomerase I by small-molecule inhibitors. Antimicrob. Agents Chemother..

[27] Godbole A.A., Ahmed W., Bhat R.S., Bradley E.K., Ekins S., Nagaraja V. (2014). Inhibition of Mycobacterium tuberculosis topoisomerase I by m-AMSA, a eukaryotic type II topoisomerase poison. Biochem. Biophys. Res. Commun..

[28] Ekins S., Godbole A.A., Kéri G., Orfi L., Pato J., Bhat R.S., Verma R., Bradley E.K., Nagaraja V. (2017). Machine learning and docking models for Mycobacterium tuberculosis topoisomerase I. Tuberculosis.

[29] (2024). The Atomwise AIMS Program. AI is a viable alternative to high throughput screening: A 318-target study. Sci. Rep..

[30] Alkan C., O’Brien T., Kenyon V., Ikegami T. (2024). Computer-Selected Antiviral Compounds: Assessing In Vitro Efficacies against Rift Valley Fever Virus. Viruses.

[31] Chen J., Bolhuis D.L., Laggner C., Kong D., Yu L., Wang X., Emanuele M.J., Brown N.G., Liu P. (2023). AtomNet-Aided OTUD7B Inhibitor Discovery and Validation. Cancers.

[32] Parijat P., Attili S., Hoare Z., Shattock M., Kenyon V., Kampourakis T. (2023). Discovery of a novel cardiac-specific myosin modulator using artificial intelligence-based virtual screening. Nat. Commun..

[33] Stecula A., Hussain M.S., Viola R.E. (2020). Discovery of Novel Inhibitors of a Critical Brain Enzyme Using a Homology Model and a Deep Convolutional Neural Network. J. Med. Chem..

[34] Hsieh C.H., Li L., Vanhauwaert R., Nguyen K.T., Davis M.D., Bu G., Wszolek Z.K., Wang X. (2019). Miro1 Marks Parkinson’s Disease Subset and Miro1 Reducer Rescues Neuron Loss in Parkinson’s Models. Cell Metab..

[35] Huang C., Bernard D., Zhu J., Dash R.C., Chu A., Knupp A., Hakey A., Hadden M.K., Garmendia A., Tang Y. (2020). Small molecules block the interaction between porcine reproductive and respiratory syndrome virus and CD163 receptor and the infection of pig cells. Virol. J..

[36] Su S., Chen J., Jiang Y., Wang Y., Vital T., Zhang J., Laggner C., Nguyen K.T., Zhu Z., Prevatte A.W. (2021). SPOP and OTUD7A Control EWS-FLI1 Protein Stability to Govern Ewing Sarcoma Growth. Adv. Sci..

[37] Wallach I., Dzamba M., Heifets A. (2015). AtomNet: A deep convolutional neural network for bioactivity prediction in structure-based drug discovery. arXiv.

[38] Friesner R.A., Banks J.L., Murphy R.B., Halgren T.A., Klicic J.J., Mainz D.T., Repasky M.P., Knoll E.H., Shelley M., Perry J.K. (2004). Glide: A new approach for rapid, accurate docking and scoring. 1. Method and assessment of docking accuracy. J. Med. Chem..

[39] Friesner R.A., Murphy R.B., Repasky M.P., Frye L.L., Greenwood J.R., Halgren T.A., Sanschagrin P.C., Mainz D.T. (2006). Extra precision glide: Docking and scoring incorporating a model of hydrophobic enclosure for protein-ligand complexes. J. Med. Chem..

[40] Tiwari P.B., Chapagain P.P., Seddek A., Annamalai T., Uren A., Tse-Dinh Y.C. (2020). Covalent Complex of DNA and Bacterial Topoisomerase: Implications in Antibacterial Drug Development. ChemMedChem.

[41] Seddek A., Madeira C., Annamalai T., Mederos C., Tiwari P.B., Welch A.Z., Tse-Dinh Y.-C. (2022). A Yeast-Based Screening System for Differential Identification of Poisons and Suppressors of Human Topoisomerase I. Front. Biosci..

[42] López-Lázaro M., Willmore E., Austin C.A. (2010). The dietary flavonoids myricetin and fisetin act as dual inhibitors of DNA topoisomerases I and II in cells. Mutat. Res..

[43] Seddek A., Annamalai T., Tse-Dinh Y.C. (2021). Type IA Topoisomerases as Targets for Infectious Disease Treatments. Microorganisms.

[44] García M.T., Carreño D., Tirado-Vélez J.M., Ferrándiz M.J., Rodrigues L., Gracia B., Amblar M., Ainsa J.A., de la Campa A.G. (2018). Boldine-Derived Alkaloids Inhibit the Activity of DNA Topoisomerase I and Growth of Mycobacterium tuberculosis. Front. Microbiol..

[45] Gupta R., Rodrigues Felix C., Akerman M.P., Akerman K.J., Slabber C.A., Wang W., Adams J., Shaw L.N., Tse-Dinh Y.C., Munro O.Q. (2018). Evidence for Inhibition of Topoisomerase 1A by Gold(III) Macrocycles and Chelates Targeting Mycobacterium tuberculosis and Mycobacterium abscessus. Antimicrob. Agents Chemother..

[46] Garcia P.K., Annamalai T., Wang W., Bell R.S., Le D., Martin Pancorbo P., Sikandar S., Seddek A., Yu X., Sun D. (2019). Mechanism and resistance for antimycobacterial activity of a fluoroquinophenoxazine compound. PLoS ONE.

[47] Gillman P.K. (2007). Tricyclic antidepressant pharmacology and therapeutic drug interactions updated. Br. J. Pharmacol..

[48] Arora G., Tiwari P., Mandal R.S., Gupta A., Sharma D., Saha S., Singh R. (2014). High throughput screen identifies small molecule inhibitors specific for Mycobacterium tuberculosis phosphoserine phosphatase. J. Biol. Chem..

[49] Ferdous S., Dasgupta T., Annamalai T., Tan K., Tse-Dinh Y.C. (2023). The interaction between transport-segment DNA and topoisomerase IA-crystal structure of MtbTOP1 in complex with both G- and T-segments. Nucleic Acids Res..

[50] Kenyon V., Chorny I., Carvajal W.J., Holman T.R., Jacobson M.P. (2006). Novel human lipoxygenase inhibitors discovered using virtual screening with homology models. J. Med. Chem..

