# Supplementary material for: Small Molecule Inhibitors of Mycobacterium tuberculosis Topoisomerase I Identified by Machine Learning and In Vitro Assays
Source: Int J Mol Sci. 2024 Nov 15;25(22):12265. doi: 10.3390/ijms252212265 (PMC11594364; doi:10.3390/ijms252212265)
Supplement: Supplementary file 1 [file ijms-25-12265-s001.zip › Figure S1.pdf]

Figure S1. Assay of inhibition of MtbTOP1 relaxation of negatively supercoiled DNA by AW-26 analogs. Results are shown for two-fold serial dilutions from 100 to 6.25 µM (A1-A6, A11) or 200 to 12.5 µM (A7, A9, A10, A12). A previously identified inhibitor of bacterial topoisomerase I (NSC76027) [40] at 8 µM was included as positive control.

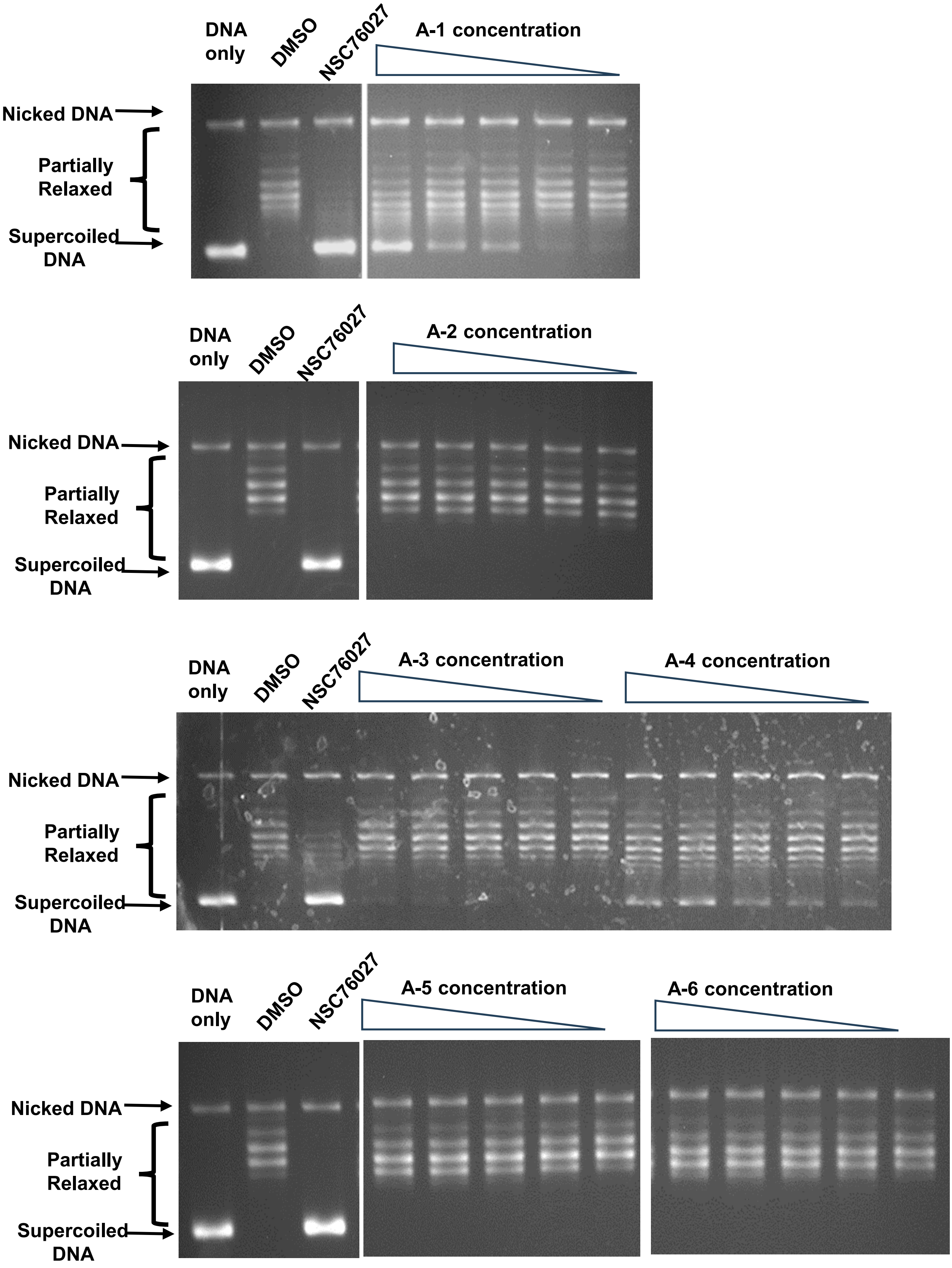

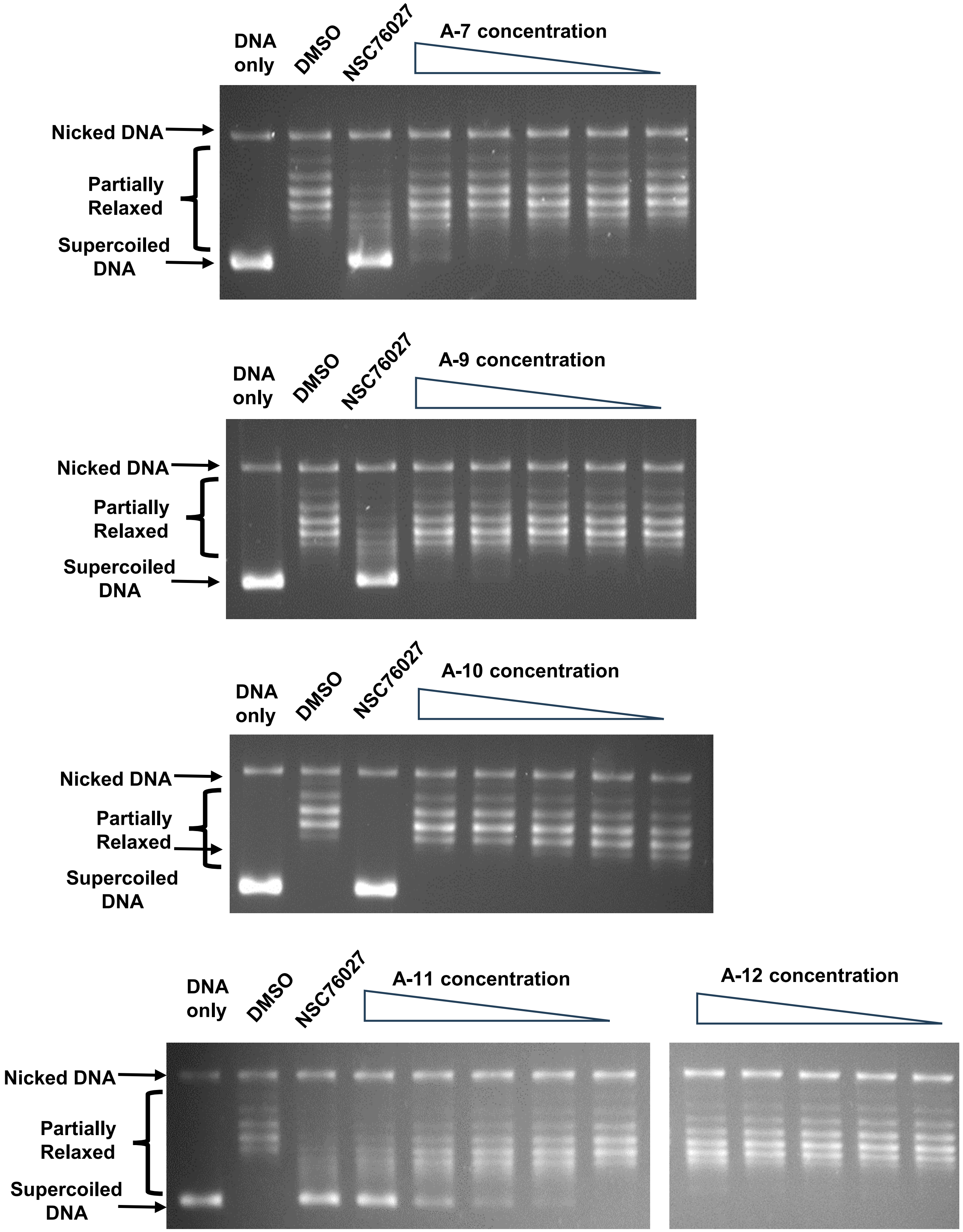

The compounds purchased from Enamine and Chembridge have minimum purity of 90% and identity confirmed using 1H-NMR and/or LC-MS..
