# Supplementary material for: Small Molecule Inhibitors of Mycobacterium tuberculosis Topoisomerase I Identified by Machine Learning and In Vitro Assays
Source: Int J Mol Sci. 2024 Nov 15;25(22):12265. doi: 10.3390/ijms252212265 (PMC11594364; doi:10.3390/ijms252212265)
Supplement: Supplementary file 1 [file ijms-25-12265-s001.zip › Figure S2.pdf]

Figure S2. Sequence alignment of *Mycobacterium tuberculosis* topoisomerase I (MtbTOP1) and *Mycobacterium smegmatis* topoisomerase I (MsmTOP1).
According to NCI Blast analysis, the identity between the two sequences is 82%. The MtbTOP1 AIM screening target site residues are denoted by the red asterisks.

```
                                   ***  *
MtbTOP1    1  MADPKTKGRGSGGNGSGRRLVIVESPTKAR   30
              MA      RGSGG G  RRLVIVESPTKAR
MsmTOP1    1  MAGGD---RGSGGTGNVRRLVIVESPTKAR   27

                           ****
MtbTOP1   31  KLASYLGSGYIVESSRGHIRDLPRAASDVP   60
              K A YLGS Y VESSRGHIRDLPR A DVP
MsmTOP1   28  KIAGYLGSNYVVESSRGHIRDLPRNAADVP   57

MtbTOP1   61  AKYKSQPWARLGVNVDADFEPLYIISPEKR   90
              AK KS PWARLGVNVD  FEPLYI SPEK
MsmTOP1   58  AKFKSEPWARLGVNVDQNFEPLYIVSPEKK   87

                                *******
MtbTOP1   91  STVSELRGLLKDVDELYLATDGDREGEAIA  120
              STV EL GLLKDVDELYLATDGDREGEAIA
MsmTOP1   88  STVTELKGLLKDVDELYLATDGDREGEAIA  117

MtbTOP1  121  WHLLETLKPRIPVKRMVFHEITEPAIRAAA  150
              WHLLETLKPR PVKRMVFHEITEPAIR AA
MsmTOP1  118  WHLLETLKPRVPVKRMVFHEITEPAIRNAA  147

                       *
MtbTOP1  151  EHPRDLDIDLVDAQETRRILDRLYGYEVSP  180
              E PRDLDI LVDAQETRRILDRLYGYEVSP
MsmTOP1  148  ENPRDLDIALVDAQETRRILDRLYGYEVSP  177

MtbTOP1  181  VLWKKVAPKLSAGRVQSVATRIIVARERDR  210
              VLWKKVAPKLSAGRVQSVATRIIV RER R
MsmTOP1  178  VLWKKVAPKLSAGRVQSVATRIIVQRERER  207

MtbTOP1  211  MAFRSAAYWDILAKLDASVSDPDAAPPTFS  240
              MAF SA YWD  A LDASVSDP A PP F
MsmTOP1  208  MAFHSASYWDVTAELDASVSDPSASPPKFT  237

MtbTOP1  241  ARLTAVAGRRVATGRDFDSLGTLRKGDEVI  270
              A L  V GRRVATGRDFDSLG L   DEV
MsmTOP1  238  AKLNTVDGRRVATGRDFDSLGQLKRPDEVL  267

MtbTOP1  271  VLDEGSATALAAGLDGTQLTVASAEEKPYA  300
              VLDE SA ALA GL G QL V S E KPY
MsmTOP1  268  VLDEASAGALASGLRGAQLAVTSVEQKPYT  297

MtbTOP1  301  RRPYPPFMTSTLQQEASRKLRFSAERTMSI  330
              RRPY PFMTSTLQQEA RKLRFS ERTMSI
MsmTOP1  298  RRPYAPFMTSTLQQEAARKLRFSSERTMSI  327

                         * *
MtbTOP1  331  AQRLYENGYITYMRTDSTTLSESAINAART  360
              AQRLYENGYITYMRTDSTTLSESAINAART
MsmTOP1  328  AQRLYENGYITYMRTDSTTLSESAINAART  357
```

```
                                         **
MtbTOP1  361  QARQLYGDEYVAPAPRQYTRKVKNAQEAHE  390
              QARQLYG EYV P PRQYTRKVKNAQEAHE
MsmTOP1  358  QARQLYGEEYVHPSPRQYTRKVKNAQEAHE  387

MtbTOP1  391  AIRPAGETFATPDAVRRELDGPNIDDFRLY  420
              AIRPAG  F TP      LD    D FRLY
MsmTOP1  388  AIRPAGDVFQTPGQLHSALDT---DEFRLY  414

MtbTOP1  421  ELIWQRTVASQMADARGMTLSLRITGM-SG  449
              ELIWQRTVASQMADARG TLSLRI G  S
MsmTOP1  415  ELIWQRTVASQMADARGTTLSLRIGGSASS  444

MtbTOP1  450  HQEVVFSATGRTLTFPGFLKAYVETVDELV  479
                 VVF A GRT TFPGFLKAYVE  DEL
MsmTOP1  445  GEQVVFNASGRTITFPGFLKAYVESIDELA  474

MtbTOP1  480  GGEADDAERRLPHLTPGQRLDIVELTPDGH  509
              GGE DDAE RLP LT GQR D   L  DGH
MsmTOP1  475  GGESDDAESRLPNLTQGQRVDAADLSADGH  504

                                       *
MtbTOP1  510  ATNPPARYTEASLVKALEELGIGRPSTYSS  539
               T PPARYTEASL KALEELGIGRPSTYSS
MsmTOP1  505  QTSPPARYTEASLIKALEELGIGRPSTYSS  534

MtbTOP1  540  IIKTIQDRGYVHKKGSALVPSWVAFAVTGL  569
              IIKTIQDRGYV KKGSALVPSWVAFAV GL
MsmTOP1  535  IIKTIQDRGYVQKKGSALVPSWVAFAVVGL  564

MtbTOP1  570  LEQHFGRLVDYDFTAAMEDELDEIAAGNER  599
              LEQHFGRLVDYDFTAAMEDELDEIA G E
MsmTOP1  565  LEQHFGRLVDYDFTAAMEDELDEIANGQEQ  594

MtbTOP1  600  RTNWLNNFYFGGDHGVPDSVARSGGLKKLV  629
              RTNWLNNFYFGG HGV  S AR GGLK LV
MsmTOP1  595  RTNWLNNFYFGGEHGVEGSIARAGGLKQLV  624

MtbTOP1  630  GINLEGIDAREVNSIKLFDDTHGRPIYYRV  659
              G NLEGIDAREVNSIK FDD  GRP YYRV
MsmTOP1  625  GGNLEGIDAREVNSIKVFDDSEGRPVYYRV  654

MtbTOP1  660  GKNGPYLERLV--AGDTGEPTPQRANLSDS  687
              G NGPYLER V      GE  PQRANL
MsmTOP1  655  GRNGPYLERMVDDPDNPGEQKPQRANLKED  684

MtbTOP1  688  ITPDELTLQVAEELFATPQQGRTLGLDPET  717
               TPDELT   AE LFATPQ GR LG DPET
MsmTOP1  685  LTPDELTPELAEKLFATPQEGRSLGIDPET  714
```

```
MtbTOP1   718  GHEIVAREGRFGPYVTEILPEP--AADAAA  745
               GHEIVA  GRFGPYVTE LPEP    D
MsmTOP1   715  GHEIVAKDGRFGPYVTEVLPEPEDGGDDGT  744

MtbTOP1   746  AAQGVKKRQKAAGPKPRTGSLLRSMDLQTV  775
               A    KK  K  GPKPRTGSL RSMDL TV
MsmTOP1   745  AGTPAKKGKKPTGPKPRTGSLFRSMDLETV  774

MtbTOP1   776  TLEDALRLLSLPRVVGVDPASGEEITAQNG  805
               TLEDAL LLSLPRVVGVDP   EEITAQNG
MsmTOP1   775  TLEDALKLLSLPRVVGVDPTTNEEITAQNG  804

MtbTOP1   806  RYGPYLKRGNDSRSLVTEDQIFTITLDEAL  835
               RYGPYLKRG DSRSL TEDQIFTITLDEAL
MsmTOP1   805  RYGPYLKRGTDSRSLATEDQIFTITLDEAL  834

MtbTOP1   836  KIYAEPKRRGRQSASAPPLRELGTDPASGK  865
               KIYAEPKRRGRQ ASAPPLRELG DP SGK
MsmTOP1   835  KIYAEPKRRGRQAASAPPLRELGNDPVSGK  864

MtbTOP1   866  PMVIKDGRFGPYVTDGETNASLRKGDDVAS  895
               PMVIKDGRFGPYVTDGETNASLRKGDDV
MsmTOP1   865  PMVIKDGRFGPYVTDGETNASLRKGDDVLT  894

MtbTOP1   896  ITDERAAELLADRRARGPAKR--PARKAAR  923
               ITDERA ELLADRRARGP K   PA KAA
MsmTOP1   895  ITDERASELLADRRARGPVKKKAPAKKAAK  924

MtbTOP1   924  KVPAKKAAKRD-  934
               K PAKKAA
MsmTOP1   925  KAPAKKAAAKKA  936
```
