# Supplementary material for: Small Molecule Inhibitors of Mycobacterium tuberculosis Topoisomerase I Identified by Machine Learning and In Vitro Assays
Source: Int J Mol Sci. 2024 Nov 15;25(22):12265. doi: 10.3390/ijms252212265 (PMC11594364; doi:10.3390/ijms252212265)
Supplement: Supplementary file 1 [file ijms-25-12265-s001.zip › Table S1.pdf]

Table S1. Enamine ID and structure of the 96 virtual screening hits

Code	Enamine ID	Structure
AW-1	Z1213658485	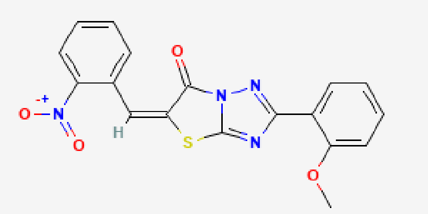
AW-2	Z87600393	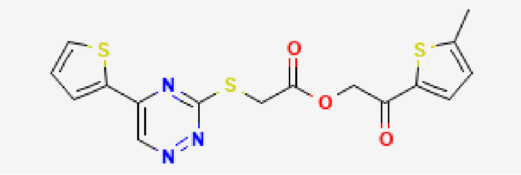
AW-3	Z168879682	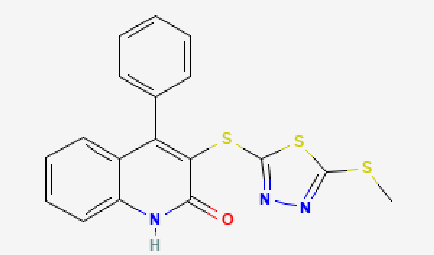
AW-4	Z98178385	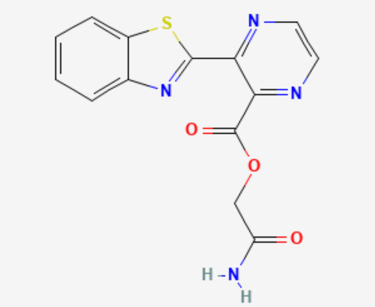
AW-5	Z242274650	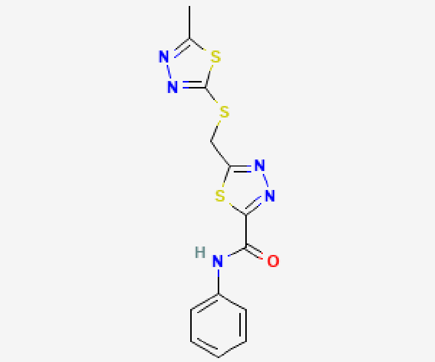
AW-6	Z271775552	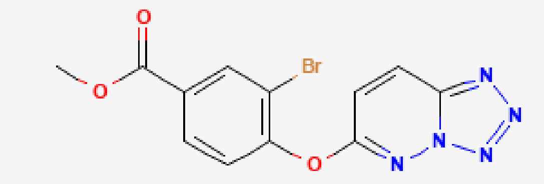

AW-7	Z324260754	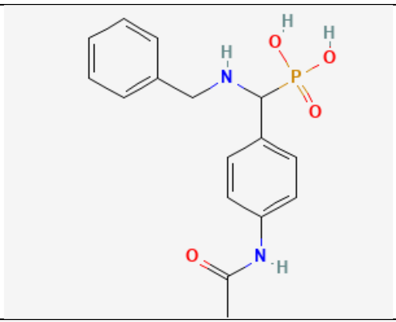
AW-8	Z113583514	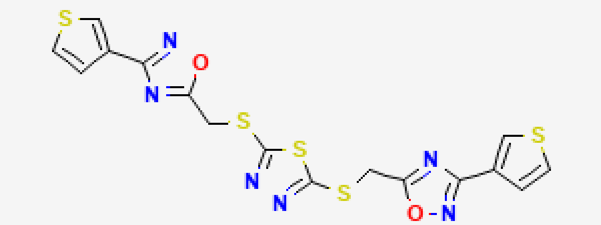
AW-9	Z17328664	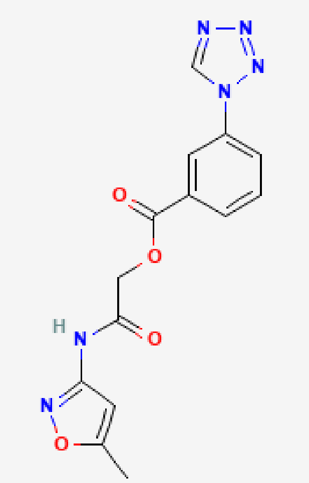
AW-10	Z73997630	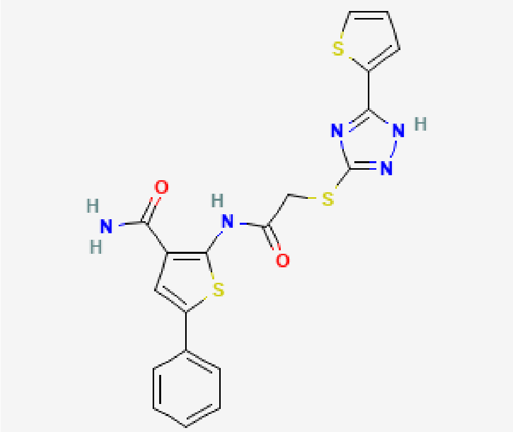
AW-11	Z1980481415	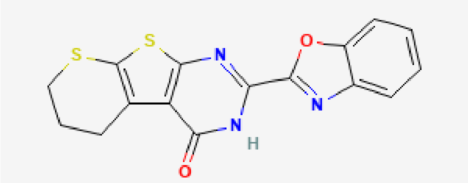

AW-12	Z56857241	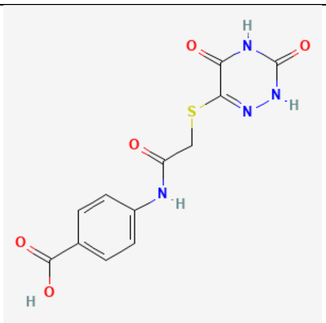
AW-13	Z96549981	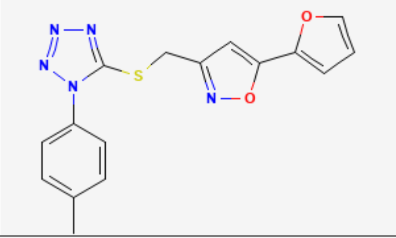
AW-14	Z113750338	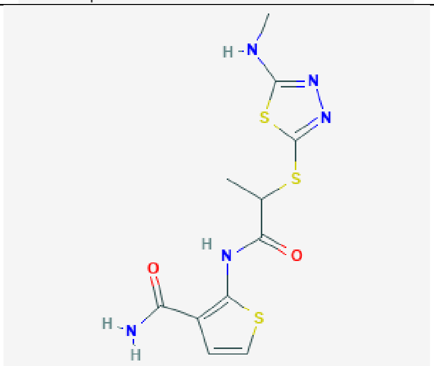
AW-15	Z1910873532	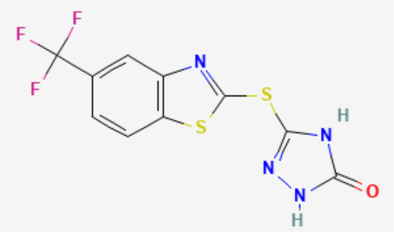
AW-16	Z952982046	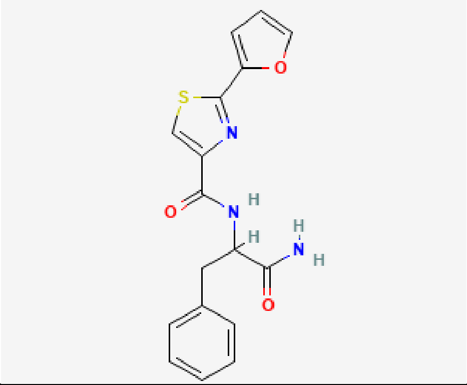

AW-17	Z92708736	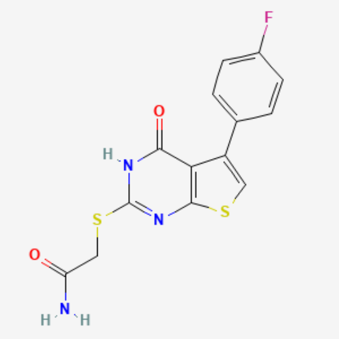
AW-18	Z1996812322	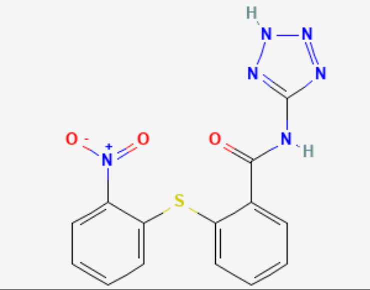
AW-19	Z44425542	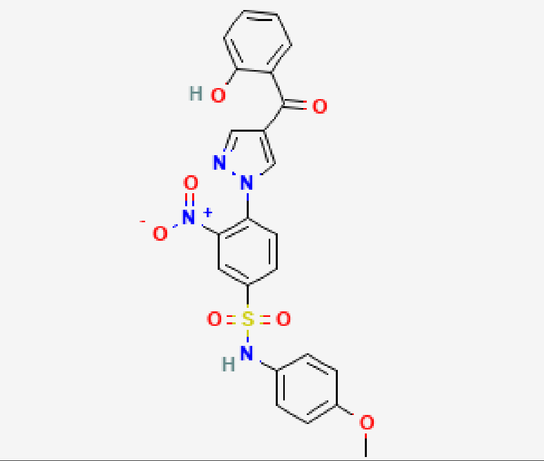
AW-20	Z18490043	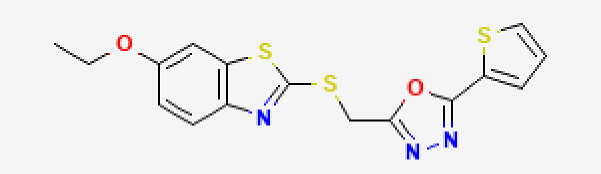
AW-21	Z369730874	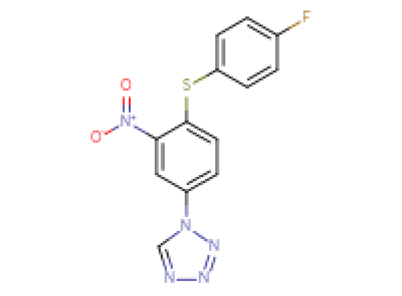

AW-22	Z56989655	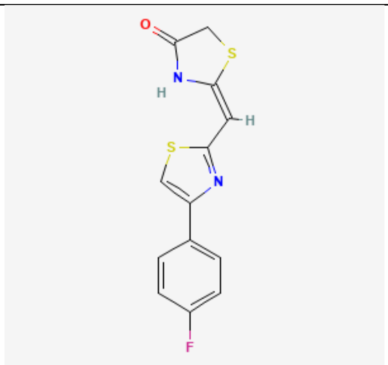
AW-23	Z55429060	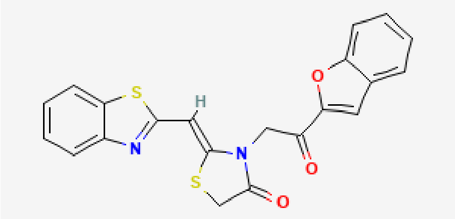
AW-24	Z57788727	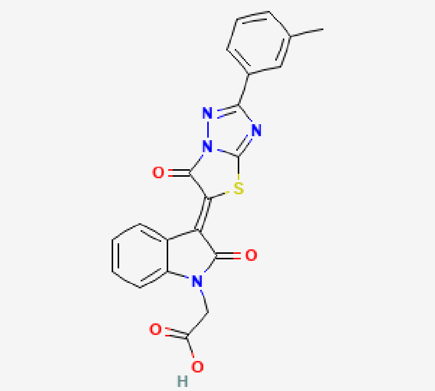
AW-25	Z57391686	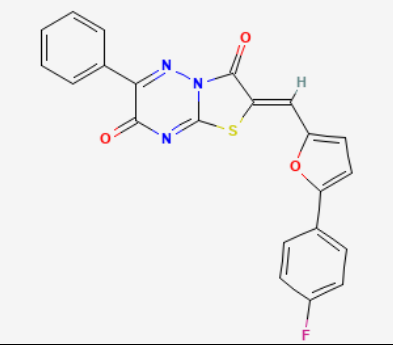
AW-26	Z15957051	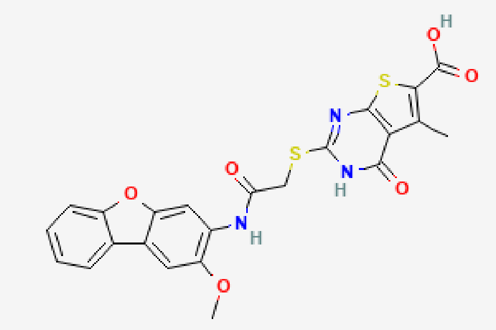

AW-27	Z126185516	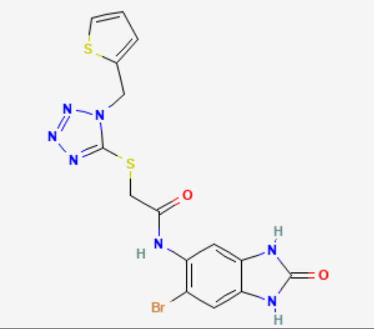
AW-28	Z19656244	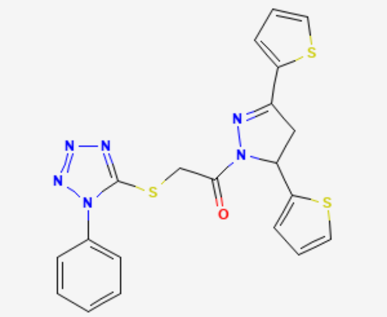
AW-29	Z89810596	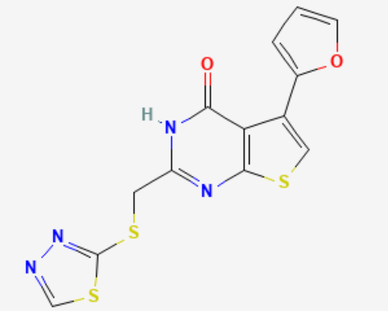
AW-30	Z166792260	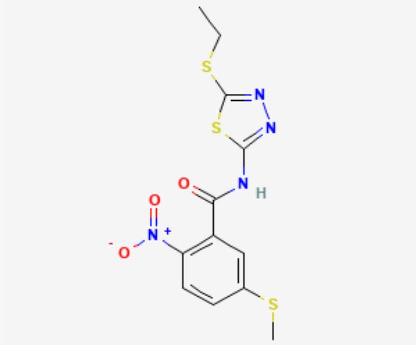
AW-31	Z87872120	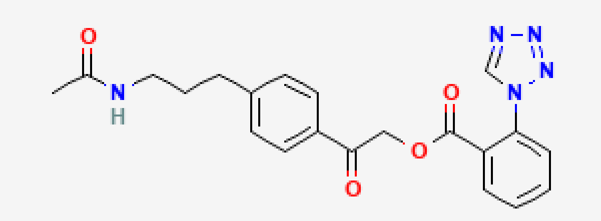

AW-32	Z56276736		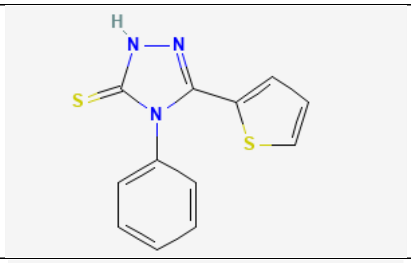
AW-33	Z160725698		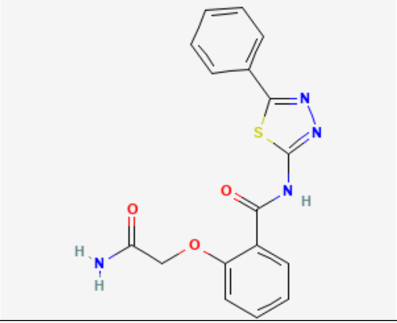
AW-34	Z295597260		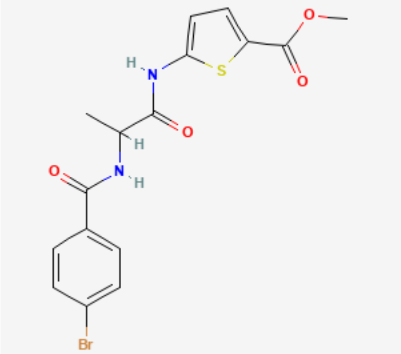
AW-35	Z25155185		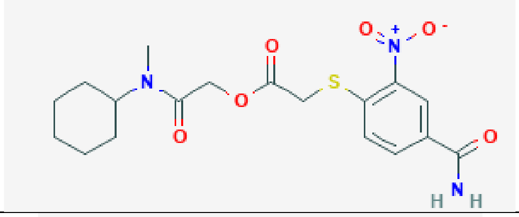
AW-36	Z228728270		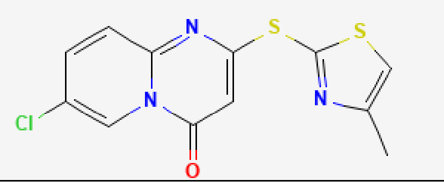
AW-37	Z97926875		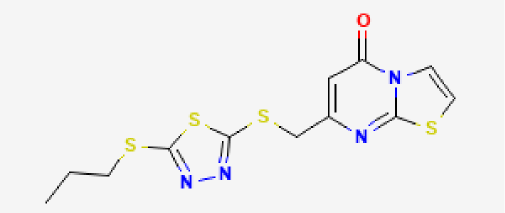

AW-38	Z116839114	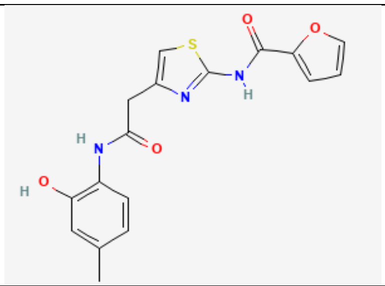
AW-39	Z226446638	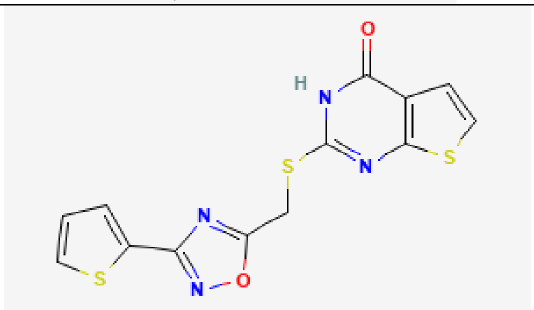
AW-40	Z229685178	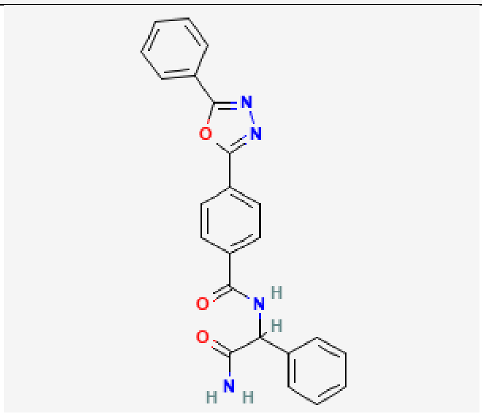
AW-41	Z238095144	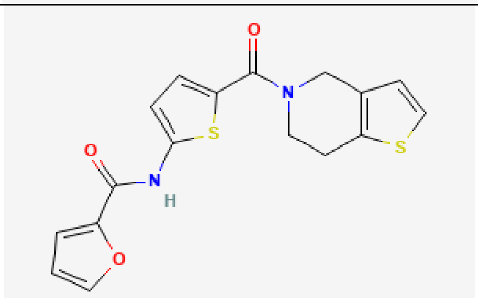
AW-42	Z368506154	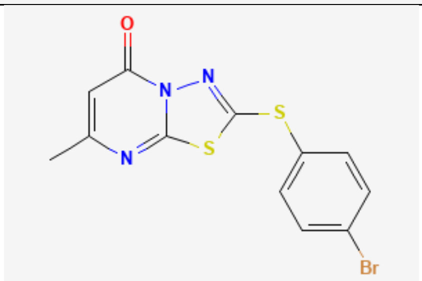

AW-43	Z89347445	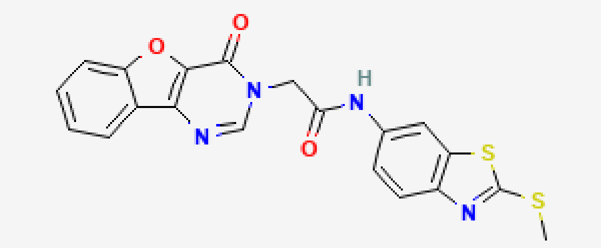
AW-44	Z185532994	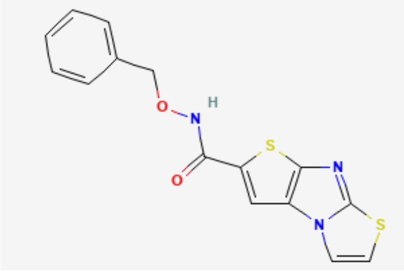
AW-45	Z1681651219	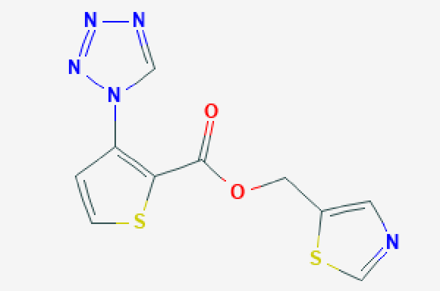
AW-46	Z317165714	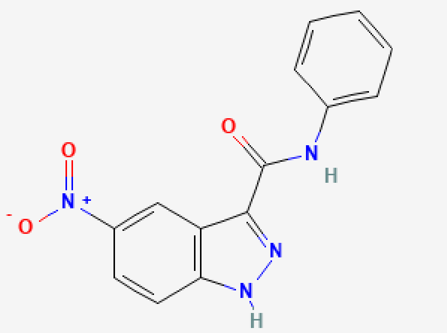
AW-47	Z905303166	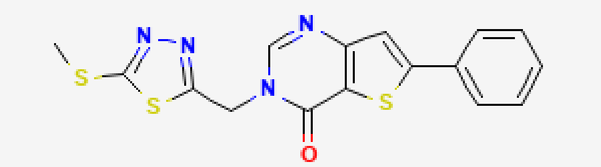
AW-48	Z381303208	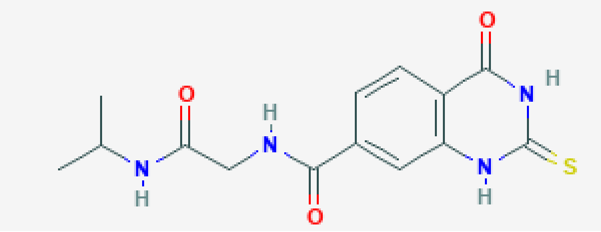

AW-49	Z1230211584	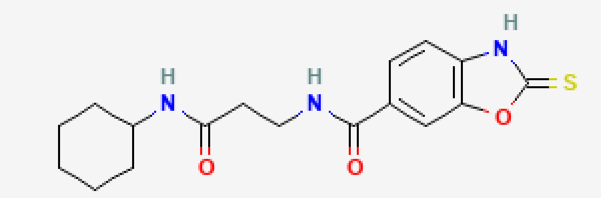
AW-50	Z220404710	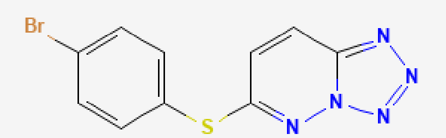
AW-51	Z335325710	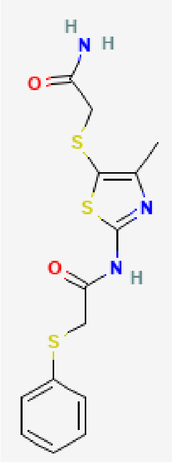
AW-52	Z735549726	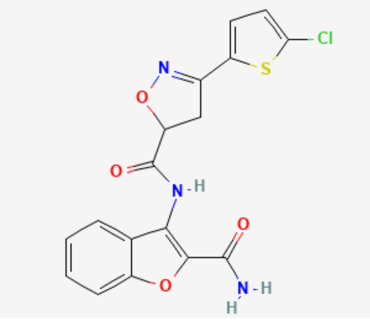
AW-53	Z1213671270	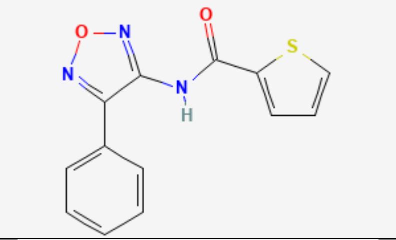
AW-54	Z89303313	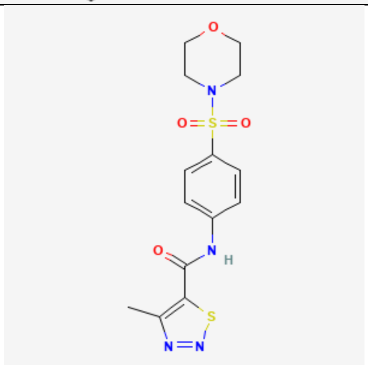

AW-55	Z19593754	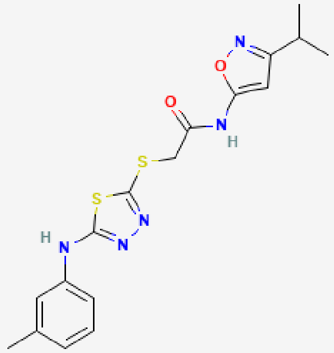
AW-56	Z56692105	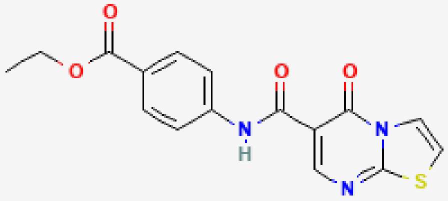
AW-57	Z318855686	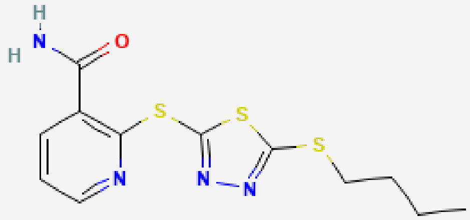
AW-58	Z1229721238	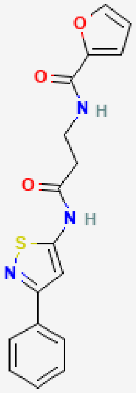
AW-59	Z154220836	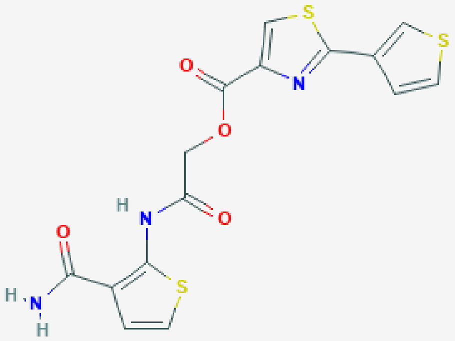

AW-60	Z599631762		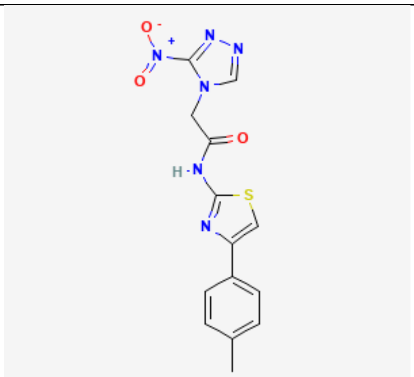
AW-61	Z90288065		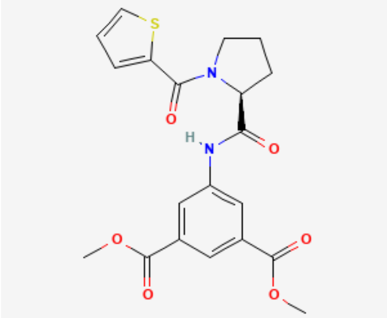
AW-62	Z57025246		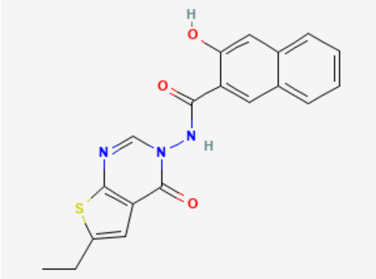
AW-63	Z18519594		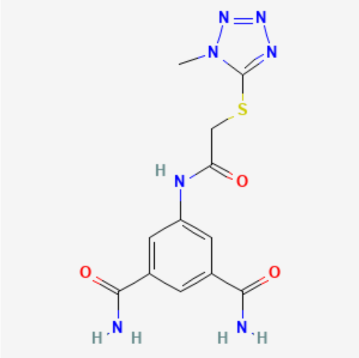
AW-64	Z57981053		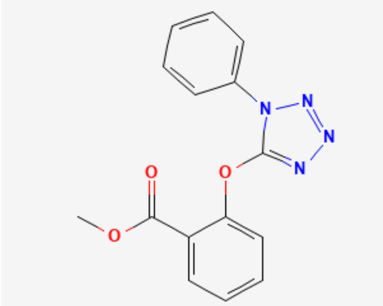

AW-65	Z606769626	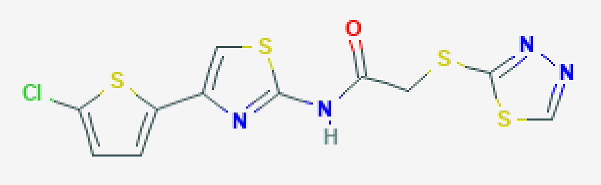
AW-66	Z1424480925	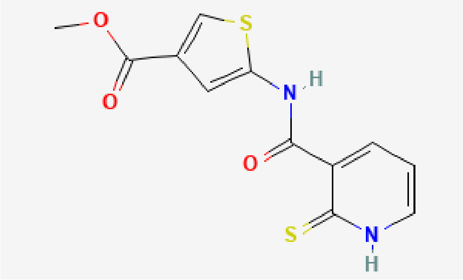
AW-67	Z27371323	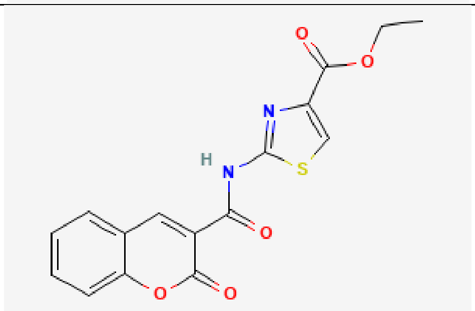
AW-68	Z18482442	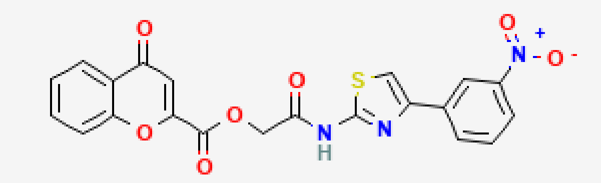
AW-69	Z18452878	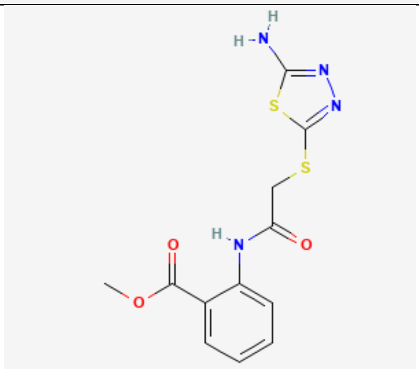
AW-70	Z153342842	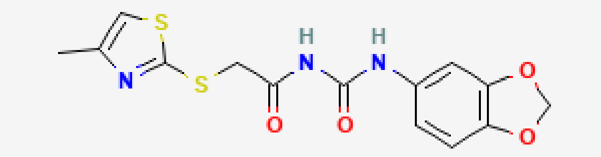

AW-71	Z24510882	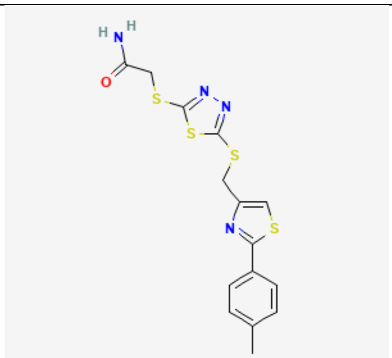
AW-72	Z1213679576	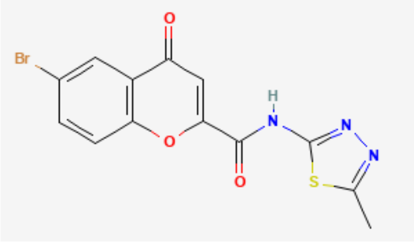
AW-73	Z19977051	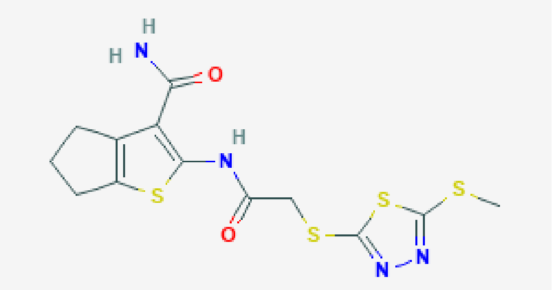
AW-74	Z85917625	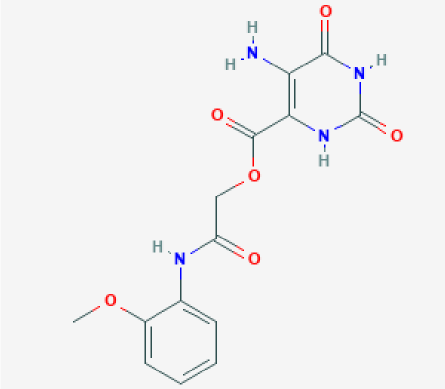
AW-75	Z56860042	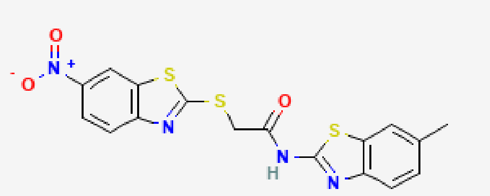
AW-76	Z1690772265	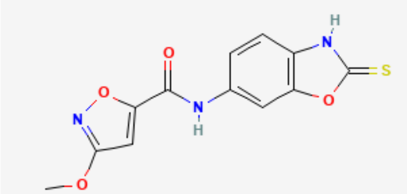

AW-77	Z56943584	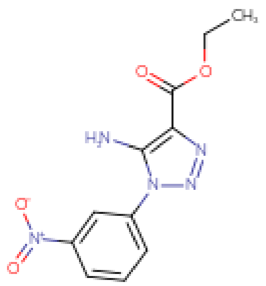
AW-78	Z224157862	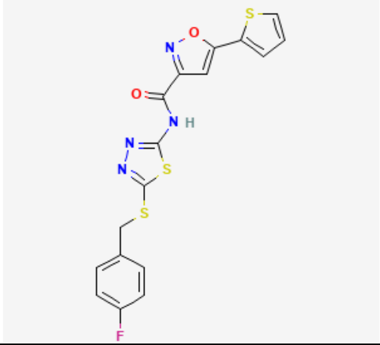
AW-79	Z1042392072	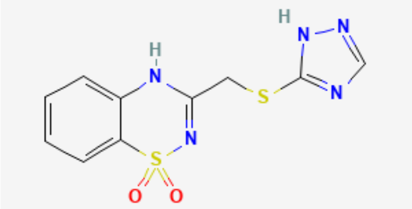
AW-80	Z221489060	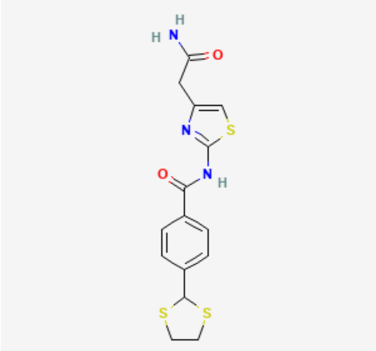
AW-81	Z1646028212	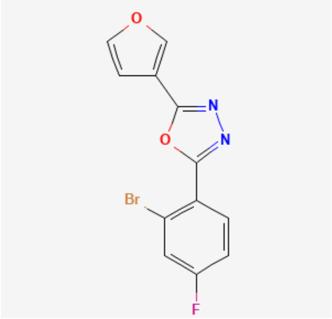

AW-82	Z1547701808		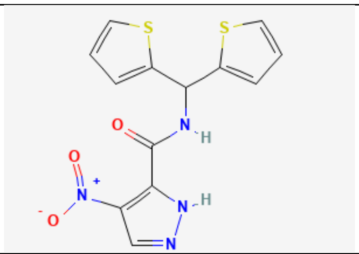
AW-83	Z1983194390		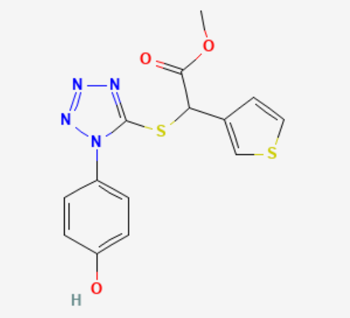
AW-84	Z751422734		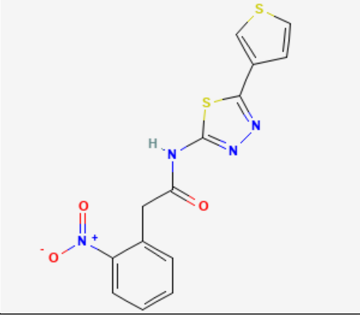
AW-85	Z1892386818		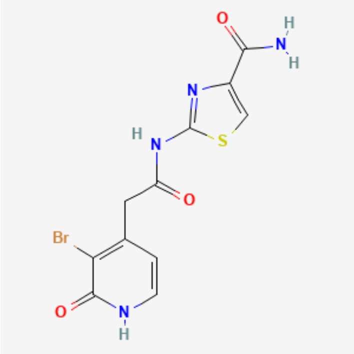
AW-86	Z2026790827		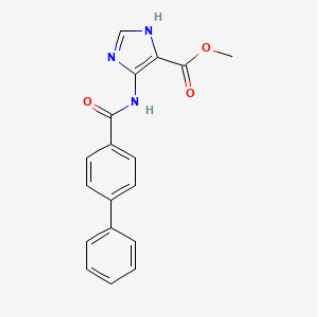

AW-87	Z3409692770		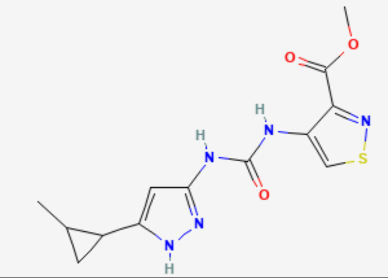
AW-88	Z2610574088		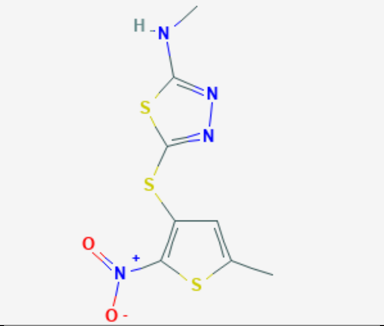
AW-89	Z324260838		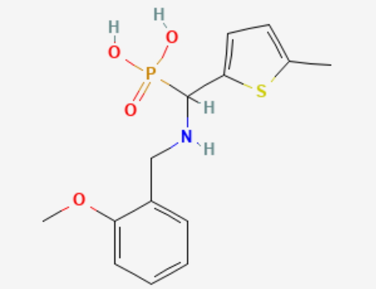
AW-90	Z335975086		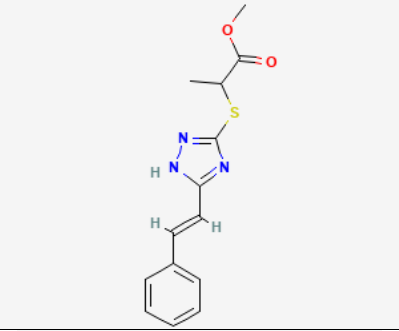
AW-91	Z369571372		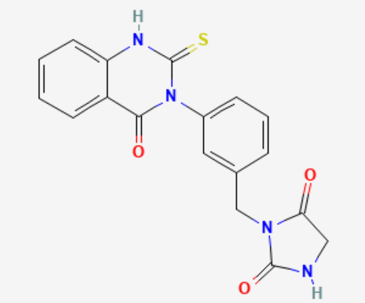

AW-92	Z1203023896	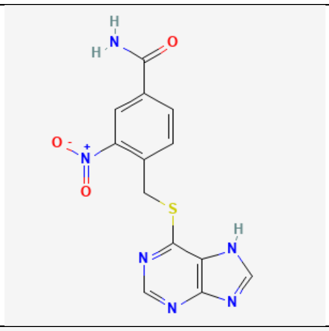
AW-93	Z991632442	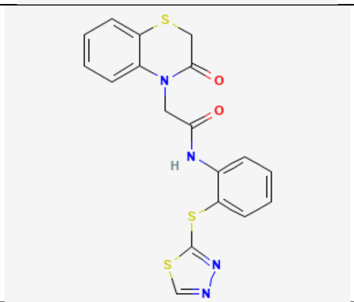
AW-94	Z19047262	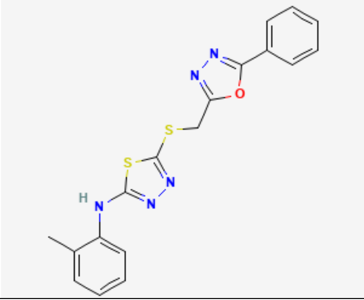
AW-95	Z32445029	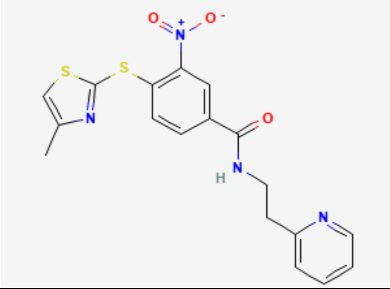
AW-96	Z441049166	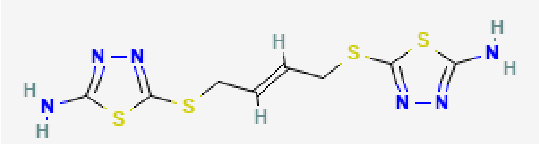
